# Discrimination of pancreatic cancer and pancreatitis by LC-MS metabolomics

**DOI:** 10.1007/s11306-017-1199-6

**Published:** 2017-04-01

**Authors:** Anna Lindahl, Rainer Heuchel, Jenny Forshed, Janne Lehtiö, Matthias Löhr, Anders Nordström

**Affiliations:** 1 Department of Molecular Biology, Umeå University, 90187 Umeå, Sweden; 2 Science for Life Laboratory, Department of Oncology-Pathology, Karolinska Institutet, Stockholm, Sweden; 3 Department of Clinical Science, Intervention and Technology (CLINTEC), Karolinska Institutet, Stockholm, Sweden

**Keywords:** Pancreatic cancer, Pancreatic ductal adenocarcinoma, Pancreatitis, Metabolomics, Metabolism, LC-MS, Biomarkers, Serum, Plasma, Discovery, Validation

## Abstract

**Introduction:**

Pancreatic ductal adenocarcinoma (PDAC) is the fifth most common cause of cancer-related death in Europe with a 5-year survival rate of <5%. Chronic pancreatitis (CP) is a risk factor for PDAC development, but in the majority of cases malignancy is discovered too late for curative treatment. There is at present no reliable diagnostic marker for PDAC available.

**Objectives:**

The aim of the study was to identify single blood-based metabolites or a panel of metabolites discriminating PDAC and CP using liquid chromatography-mass spectrometry (LC-MS).

**Methods:**

A discovery cohort comprising PDAC (n = 44) and CP (n = 23) samples was analyzed by LC-MS followed by univariate (Student’s *t* test) and multivariate (orthogonal partial least squares-discriminant analysis (OPLS-DA)) statistics. Discriminative metabolite features were subject to raw data examination and identification to ensure high feature quality. Their discriminatory power was then confirmed in an independent validation cohort including PDAC (n = 20) and CP (n = 31) samples.

**Results:**

Glycocholic acid, N-palmitoyl glutamic acid and hexanoylcarnitine were identified as single markers discriminating PDAC and CP by univariate analysis. OPLS-DA resulted in a panel of five metabolites including the aforementioned three metabolites as well as phenylacetylglutamine (PAGN) and chenodeoxyglycocholate.

**Conclusion:**

Using LC-MS-based metabolomics we identified three single metabolites and a five-metabolite panel discriminating PDAC and CP in two independent cohorts. Although further study is needed in larger cohorts, the metabolites identified are potentially of use in PDAC diagnostics.

**Electronic supplementary material:**

The online version of this article (doi:10.1007/s11306-017-1199-6) contains supplementary material, which is available to authorized users.

## Introduction

Pancreatic ductal adenocarcinoma (PDAC) is the fourth most common cause of cancer-related death with a 5-year survival rate of <5% (Siegel et al. 2016). In contrary to many other forms of cancer the incidence of PDAC is rising and predicted to become the 2nd most common cause of cancer-related death by 2030 (Rahib et al. 2014). Its lethality can be attributed to late diagnosis, almost complete resistance to conventional chemo- and radiotherapy, and a lack of diagnostic biomarkers. Unlike breast or colorectal cancer, the only treatment with curative intent is surgery, for which < 20% of patients qualify; but even then the chance of survival is very low (Lohr 2006). Therefore, PDAC should be treated as a medical emergency (Lohr 2014). Risk factors for PDAC include chronic pancreatitis (CP) and around 5% of CP patients develop PDAC over time (Pinho et al. 2014; Garrido-Laguna and Hidalgo 2015). Both PDAC and CP are characterized by an excessive and reactive stroma, or desmoplasia, which makes it difficult to distinguish between the two conditions (Kloppel and Adsay 2009). The one FDA-approved biomarker for PDAC, carbohydrate antigen 19-9 (CA 19-9), is only useful for prognosis and detection of disease recurrence (Fong and Winter 2012). Therefore there is an urgent clinical need of a non-invasive diagnostic biomarker for PDAC.

Metabolomics is the untargeted analysis of low molecular weight endogenous compounds, metabolites, present in e.g. a defined biological sample such as tissue or a bodily fluid as blood (Fiehn et al. 2000; Fiehn 2002; Spratlin et al. 2009; Dunn et al. 2011). The most widely used analytical platform for metabolomics providing the largest metabolome coverage is liquid chromatography-mass spectrometry (LC-MS) (Want et al. 2007; Patti et al. 2012; Yin and Xu 2014). Given the established connection between cell metabolism and cancer (Hanahan and Weinberg 2011), LC-MS metabolomics has been applied to identify metabolite markers for several cancers (Spratlin et al. 2009; Nordstrom and Lewensohn 2010; Nicholson et al. 2012), including PDAC (Daemen et al. 2015; Rios Peces et al. 2016). Specifically, there have been previous LC-MS based metabolomics efforts to compare PDAC and CP blood samples (Urayama et al. 2010; Fukutake et al. 2015). There is however still no screening test for PDAC available.

In the present study, we have performed LC-MS based metabolomics on blood samples to compare the metabolic profiles of PDAC and CP in two independent cohorts. Both single discriminative metabolites and a panel of metabolites discriminating PDAC and CP were identified. Our findings have potential clinical relevance for early diagnosis of PDAC among CP patients to improve patient survival.

## Materials and methods

### Clinical samples

The study comprised a discovery- and a validation cohort (Table 1). All patients had given informed consent in accordance with the Helsinki Declaration (World Med 2013).

**Table 1:** Clinical data

	Discovery cohort		Validation cohort	
	PDAC	CP	PDAC	CP
Subjects (n)	44	23	20	31
Male/female ratio	21/20^*a*^	18/5	10/10	16/15
Age, median (range)	67 (30–102)^*b*^	36 (31–93)	70 (46–80)	69 (23–87)

^*a*^n = 41,

^*b*^n = 42

Blood samples for the discovery cohort were collected from German patients with PDAC (n = 44) and patients with CP (n = 23). Serum samples were prepared at the same location using a standardized protocol.

The validation cohort included Swedish PDAC patients (n = 20) and CP patients (n = 31). Plasma samples were supplied by the Karolinska Institutet biobank and had been prepared at different sites following a standardized procedure: whole blood samples were obtained at fasting early in the morning, collected in sodium citrate tubes and immediately placed in −80 °C.

All plasma and serum samples were stored at −80 °C at all times prior to analysis.

### Sample preparation

All solvents used below were of HPLC grade (Fisher Chemicals) and the water was Milli-Q (Millipore).

Plasma and serum samples were thawed on ice and 50 µl aliquots were mixed with 150 µl MeOH for protein precipitation. Samples were then centrifuged for 15 min at 15800×g. The supernatant was transferred to a new tube and then evaporated to complete dryness in a vacuum concentrator. Prior to LC-MS analysis, samples were reconstituted in 50 µl MeOH:H_2_O 1:1 and the two isotopically labelled internal standards phenylalanine (D5) and palmitic acid (D4) (Cambridge Isotope Laboratories) were added at a final concentration of 5 µM.

### LC-MS analysis

Metabolites were separated by reversed phase liquid chromatography and detected by electrospray ionization (ESI) mass spectrometry in positive mode. All samples were run in randomized order, randomized by the random number function in Excel (Microsoft, USA).

For the discovery cohort, the analytical platform consisted of a 1200 HPLC-system (Agilent) connected on-line to an LTQ Orbitrap Velos equipped with a HESI probe (Thermo Scientific). 5 µl of each sample were injected onto a Kinetex C18 150 × 2.1 mm column, 2.6 µ, 100 Å (Phenomenex) and the following mobile phases were used at a flow rate of 0.4 ml/min: H_2_O with 0.1% formic acid (A) and 3:1 acetonitrile:isopropanol with 0.1% formic acid (B). The linear gradient was 0 min, 5% B; 1 min, 5% B; 21 min, 95% B; 26 min, 95% B; 26.5 min, 5% B; 33 min, 5% B. MS data was collected in centroid mode between m/z 80-1200 at an Orbitrap resolution of 60,000 using the following ESI settings: Cap Temp, 350; Sheath Gas Flow, 45; Aux Gas Flow, 15; Source Voltage, 3 kV; S-Lens RF Level, 60%.

For the validation cohort, the analytical platform consisted of a 1290 UHPLC-system (Agilent) connected on-line to a 6550 Q-ToF equipped with a JetStream source (Agilent). 5 µl of each sample were injected onto a Kinetex C18 100 × 2.1 mm column, 2.6 µ, 100 Å (Phenomenex) and the following mobile phases were used at a flow rate of 0.5 ml/min: H2O with 0.1% formic acid (A) and 3:1 acetonitrile:isopropanol with 0.1% formic acid (B). The linear gradient was 0 min, 5% B; 7 min, 95% B; 9 min, 95% B; 9.2 min, 5% B; 11 min, 5% B. MS data was collected in centroid mode between m/z 70-1700 using the following ESI settings: Gas Temp, 300; Gas Flow, 8; Nebulizer Pressure, 40; Sheet Gas Temp, 350; Sheet Gas Flow, 11; Vcap, 4000; Fragmentor, 100; Skimmer1, 45; OctapoleRFPeak, 750. The analysis procedure has also been described previously (Staubert et al. 2015; Lindahl et al. 2016).

The peak heights for the internal standards were continuously monitored during analysis to ensure MS signal stability (Supplementary table 1). In the validation cohort the signal stability was monitored by injection of a pooled QC sample for every sixth sample during the LC-MS analysis. Examples of extracted ion chromatograms are illustrated in Supplementary Fig. 1.

Targeted MS/MS data of a pooled sample was also collected for identification purposes of discriminative features in both cohorts using the ESI settings described above (Supplementary Fig. 2). Samples were analyzed on two different instruments for reasons of instrument availability at the time of receiving the different cohorts to the laboratory.

### Discovery cohort data preprocessing

Discovery raw data files were converted to .cdf format using the file converter tool in Xcalibur version 2.2 (Thermo Scientific). Peak detection, integration and alignment were performed with the open-source software XCMS version 1.30.3 (Smith et al. 2006). The following XCMS settings were used: For peak detection and integration with the centWave algorithm, ppm = 10, snthr = 5, peakwidth = c(5.15), mzdiff = −0.01, prefilter = c(6,100), fitgauss = TRUE; for alignment with Obiwarp, distFunc = “cor”, profStep = 1, grouping parameters bw = 1, mzwid = 0.005, minfrac = 0.5. Isotopes and adducts were not removed.

Sample-wise median normalization (Trezzi et al. 2015) was used to correct for technical variation in e.g. sample preparation and MS signal intensity. Next, a two-step peak filtering approach was applied. First, peaks with an intensity level ≥ 60,000 were retained. Second, only peaks present in at least 75% of samples in at least one group were used for the following statistical analyses.

### Univariate analysis

**Fig. 1:**
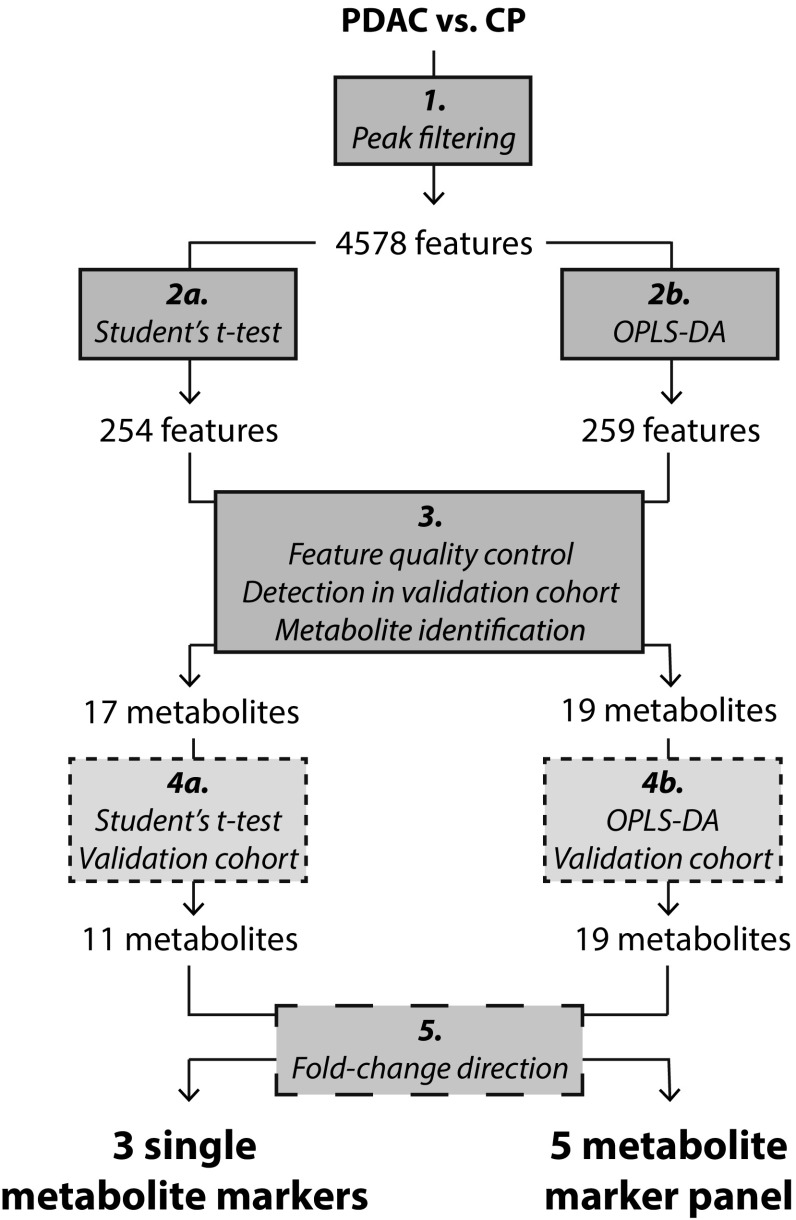
Schematic overview of data analysis workflow. Univariate (*left*) and multivariate (*right*) analyses comparing PDAC and CP were performed in parallel, starting with the abundance filtered discovery cohort data set (step 1). The large number of metabolite features (n = 4578) is due to superfluous features e.g. adducts and isotopes. Following statistical analysis of the discovery cohort (step 2a and b), selected metabolite features were examined manually in the raw data and low quality features (e.g. adducts, isotopes, low intensity peaks) were excluded (step 3). High quality features that were not present in the validation cohort were excluded as well, together with features that could not be identified. The discriminative capacity of the remaining identified features, now referred to as metabolites, was confirmed in the validation cohort by statistical analysis (step 4a and b). Finally, metabolites that were regulated in opposite directions in the two cohorts were excluded (step 5), resulting in three single metabolite markers and a panel of five metabolite markers

Both univariate and multivariate statistical analysis were applied in parallel (Fig. 1).

Univariate analysis was performed in the software GraphPad Prism version 6.07 (GraphPad), where PDAC and CP were compared using a Student’s *t* test with Welch’s correction. In the discovery cohort, metabolite features with p-value > 0.05 were excluded in order to limit the number of features to be examined in the raw data.

Box plots and bar graphs were created in GraphPad Prism.

### Multivariate analysis

For multivariate analysis, data sets were imported into the software SIMCA version 14 (Umetrics). Unit variance scaling was applied to give metabolite features with low and high variation between samples equal importance, and log transformation was applied to shift data towards Gaussian distribution. Principal component analysis (PCA) was initially applied to all variables extracted using XCMS (Supplementary Fig. 3). Subsequently was orthogonal partial least squares-discriminant analysis (OPLS-DA) (Bylesjo et al. 2006) with default SIMCA settings was used to identify metabolite feature patterns discriminating PDAC and CP. The features with the highest discriminatory power between classes were selected based on the variable importance for the projection (VIP) plot. Features significant with a 95% confidence interval, based on the cross- validation, were selected for further analysis. For the discovery cohort, the feature selection was done in the same way with the additional criterion that the VIP-values should be >1.5.

For refined OPLS-DA models, i.e. those built on selected metabolite features only, cross-validation settings were changed from the default 1/7 to 1/5 of samples constituting the internal prediction set. The purpose was to minimize the bias of the refined model towards the selected metabolite features.

All score- and loading scatter plots were created in SIMCA.

### Feature quality control

The discriminative metabolite features selected in the discovery cohort had to meet several criteria to ensure feature quality, regardless of the method of statistical analysis. First, features were manually examined in the raw data. Isotopes, adducts and poor quality features, e.g. low intensity peaks were excluded following manual inspection of peak shape. This removed features judged by the software to be chromatographic peaks, but which they human eye rapidly can assign to be column bleed or similar artefacts. Second, for the univariate part of the analysis workflow, features were re-integrated in the raw data using the Qual and Quan Browsers in Xcalibur version 2.2 (Thermo Scientific) and their statistical significance was confirmed with a Student´s *t* test (p < 0.05). Third, ion chromatograms of the selected features were extracted from the validation cohort raw data files using the software MassHunter Qual version B.06.00 (Agilent); discovery cohort features that were either not detected or of low quality in the validation cohort were excluded. Fourth, unidentifiable metabolite features were excluded.

Peak areas of the identified metabolites that met quality control criteria were integrated in the validation cohort raw data files using the “Agile” setting in MassHunter Qual. The median normalized peak area values were used for further uni- and multivariate analyses to confirm the results from the discovery cohort.

### Metabolite identification

Accurate mass measurements were subject to database searches in the public databases METLIN (Smith et al. 2005) and Human Metabolome Database (Wishart et al. 2013) as well as an in-house library comprising 384 synthetic standards. Database hits were then confirmed by retention time match (in-house library only) and MS/MS spectral match from pooled samples (Supplementary Fig. 2). In a few cases additional synthetic standard compounds were acquired and analyzed on the same platform to confirm compound identity.

For the discovery cohort, samples were also fractionated by LC and relevant fractions were then analyzed by direct infusion to acquire MS^n^ structural information (van der Hooft et al. 2012).

## Results

### Univariate analysis identifies three single metabolites discriminating PDAC and CP

**Fig. 2:**
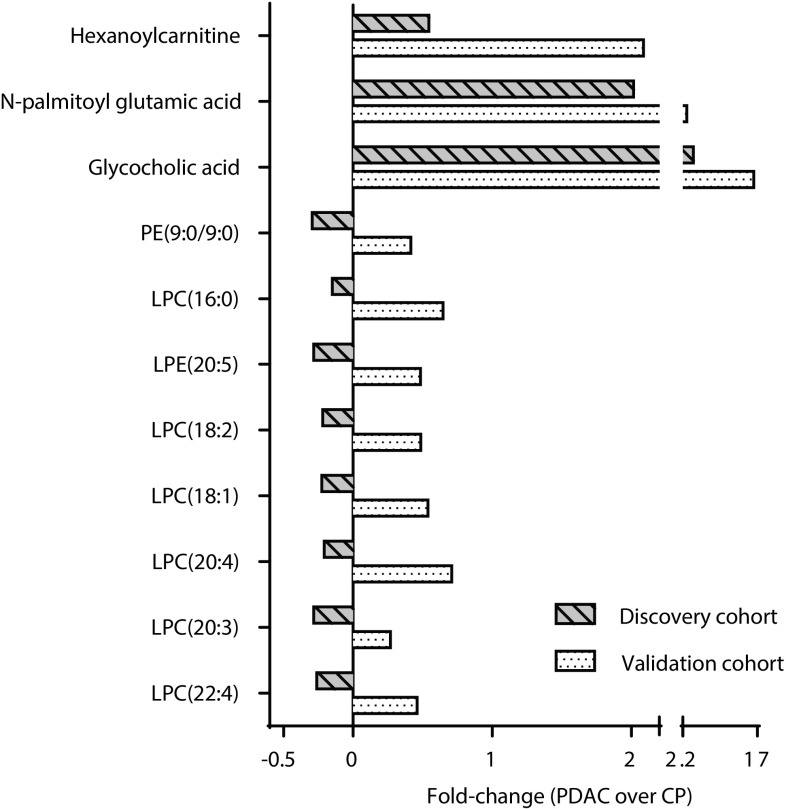
Fold-change comparison between cohorts. Following confirmation of metabolite significance in the validation cohort (Fig. 1, step 4a and b), fold-change calculations revealed that eight metabolites were regulated in opposite directions in the discovery- and validation cohorts. All eight metabolites were phospholipids that were down-regulated in PDAC compared to CP in the discovery cohort, but up-regulated in PDAC compared to CP in the validation cohort. Consequently all phospholipids were excluded as potential single metabolite markers

**Fig. 3:**
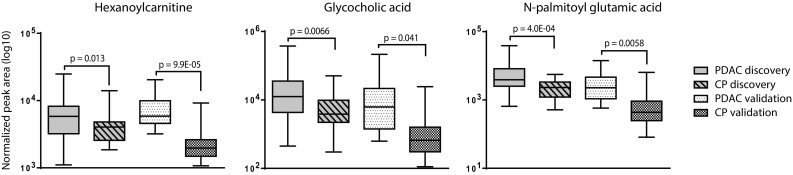
Single metabolite markers discriminating PDAC and CP. After identification of significant features in the discovery cohort (n = 67), feature quality control and confirmation of significance in the validation cohort (n = 51), three single metabolite markers for PDAC compared to CP remained. All three were up-regulated in PDAC. Statistical test: Welch’s unequal variances *t* test. *Box plot* settings: Range, minimum to maximum value; line at median

After abundance peak filtering, the discovery cohort data set contained 4578 metabolite features including isotopes, adducts and low quality features (Fig. 1). 254 of these features had a p-value < 0.05 as determined by Student’s *t* test with Welch’s correction. After *(i)* raw data examination, *(ii)* re-integration and confirmation of statistical significance, *(iii)* detection in validation cohort and *(iv)* feature identification, 17 metabolites remained. 11 of these metabolites had a p-value < 0.05 (Student’s *t* test) in the validation cohort as well. Fold-change calculations showed that the eight metabolites with the lowest intensity were down-regulated in PDAC compared to CP in the discovery cohort, as opposed to up-regulated in PDAC compared to CP in the validation cohort (Fig. 2). These eight metabolites were phospholipids. After exclusion of the phospholipids, the three metabolites glycocholic acid, hexanoylcarnitine and N-palmitoyl glutamic acid remained as single discriminative markers for PDAC compared to CP (Fig. 3). In the discovery cohort, applying an FDR cut-off of 5% to the 254 metabolites did not yield any significant metabolites. However the manual inspection and re-integration together with the validation in an independent patient cohort strengthens the findings.

### Multivariate analysis identifies a panel of five metabolites discriminating PDAC and CP

**Fig. 4:**
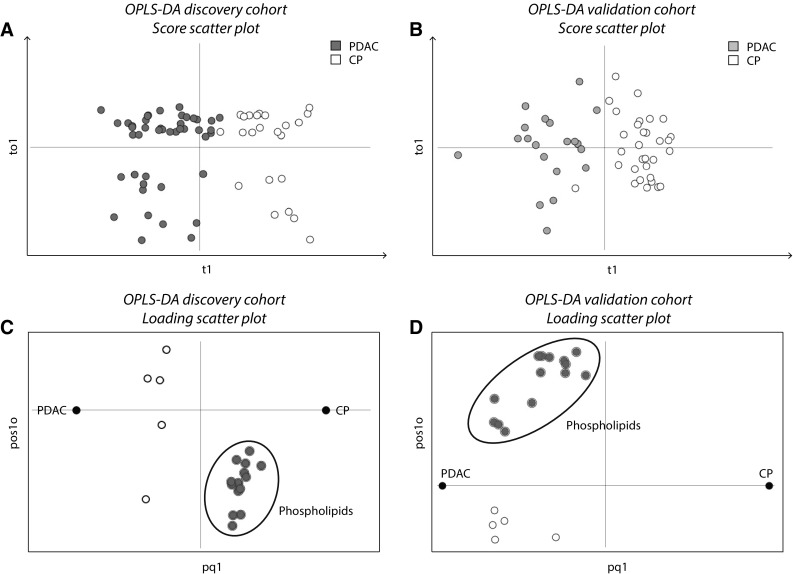
Reversed metabolite fold-change direction in discovery- and validation cohorts. **a** Score scatter plot for the initial OPLS-DA model of 4578 metabolite features in the discovery cohort (Fig. 1, step 2b). *Blue dots* represent PDAC samples (scores, n = 44); *white dots* represent CP samples (n = 23); t1 on the x-axis, first component; to1 on the y-axis, first of two orthogonal components. Model parameters: CV-groups, 7; R2X(cum) 0.39; Q2(cum) 0.22; CV-ANOVA p = 0.02. The model was used to select 259 features for further analysis. **b** Score scatter plot for the OPLS-DA model in the validation cohort based on the 19 metabolites remaining from the discovery cohort (Fig. 1, step 4b). *Orange dots* represent PDAC samples (n = 20); *white dots* represent CP samples (n = 31); to1 on the y-axis, first (and only) orthogonal component. Model parameters: CV-groups, 7; R2X(cum) 0.648; Q2(cum) 0.699; CV-ANOVA p = 1.7E-11. **c** Loading (metabolite) scatter plot for the discovery cohort OPLS-DA model. The phospholipids are situated to the right of the vertical line, close to the dummy CP variable, indicating that they are down-regulated in PDAC compared to CP. **d** Loading scatter plot for the validation cohort OPLS-DA model. The phospholipids are situated to the *left* of the vertical line, close to the dummy PDAC variable; hence they are up-regulated in PDAC compared to CP. Based on this reversal of their fold-change directions, the 14 phospholipids were excluded from the final metabolite marker panel

259 of the 4578 metabolite features in the discovery cohort (Fig. 1, step 2b) were selected as potential markers for PDAC as determined by the VIP-plot in the initial OPLS-DA model (Fig. 4a). 19 metabolites remained after feature quality control, detection in validation cohort and metabolite identification. These 19 metabolites were significant in the validation cohort OPLS-DA model (Fig. 4b). A comparison of the loading scatter plots for the discovery- and validation cohort OPLS-DA models showed that a group of 14 metabolites was regulated in opposite directions in the two cohorts (Fig. 4c, d). As in the univariate analysis, all 14 were phospholipids that were down-regulated in PDAC compared to CP in the discovery cohort but up-regulated in PDAC in the validation cohort.

**Fig. 5:**
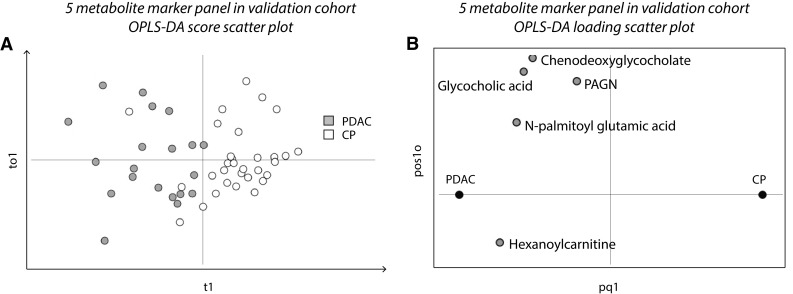
A marker panel of five metabolites discriminates PDAC and CP. **a** Score scatter plot for the refined OPLS-DA model of the five discriminative metabolites with consistent fold-change directions in the validation cohort (Fig. 1, step 4b and 5). *Orange dots* represent PDAC samples (n = 20); white dots represent CP samples (n = 31); t1 on the x-axis, first component; to1 on the y-axis, first (and only) orthogonal component. Model parameters: CV-groups, 5; R2X(cum) 0.736; Q2(cum) 0.513; CV-ANOVA p = 8.2E-07. **b** Corresponding loading scatter plot. All five metabolites in the marker panel show increased levels in PDAC compared to CP

After exclusion of the phospholipids the five metabolites N-palmitoyl glutamic acid, glycocholic acid, hexanoylcarnitine, chenodeoxyglycocholate and PAGN remained. They were used to build a refined validation cohort OPLS-DA model (Fig. 5a, b) to evaluate their discriminatory power in the absence of phospholipids. The predictive ability of the refined model as indicated by the Q2(cum) value was 0.513, i.e. approximately 50% of samples were correctly classified, and the R2X(cum) value was 0.736. The statistical significance of the model as indicated by cross-validated ANOVA was p = 8.2E−07.

## Discussion

The PDAC incidence rate is expected to increase worldwide over the next years (Rahib et al. 2014), making the need for improved diagnostic tools even more acute. One group at risk of PDAC development is CP patients (Pinho et al. 2014) who would clearly benefit from the discovery of novel markers for PDAC diagnosis.

Any preclinical biomarker study at the discovery stage should include an inflammatory control of the target organ to increase the chance of identifying disease-specific markers (Chechlinska et al. 2010; Lindahl et al. 2016). Here, CP serves as an inflammatory control of the pancreas for PDAC-specific marker discovery. In addition, CP is a risk factor for PDAC development and the two diseases share several inflammatory parameters. It is therefore imperative to identify PDAC-unique markers specific for the discrimination of PDAC and CP.

In the present study, we have compared the metabolic profiles of PDAC and CP in blood in two independent cohorts using untargeted LC-MS. The fact that both cohorts did not consist of either serum or plasma is a drawback of the study. However, that they represent completely independent patients strengthens the validity of the proposed biomarkers. Previous reports comparing the metabolite profiles of serum and plasma samples have found good correlation between the two sample matrices (Denery et al. 2011; Yu et al. 2011; Ishikawa et al. 2014). We have identified three single discriminative metabolites as well as a five-metabolite panel of markers, since a marker panel can increase specificity and sensitivity compared to single discriminative metabolites (Wang et al. 2011; Wingren et al. 2012). All five metabolites identified here can be easily measured in a clinical MS-lab.

PDAC and CP blood samples have been compared by LC-MS metabolomics analysis in two previous studies (Urayama et al. 2010; Fukutake et al. 2015). One of these targeted free amino acids in plasma. The advantage of a targeted approach is that sample preparation and analysis is optimized for a specific class of metabolites, but at the cost of covering only a limited part of the metabolome. Untargeted LC-MS on the other hand has the largest potential to identify novel metabolites due to increased metabolome coverage (Patti et al. 2012). In the second previous study comparing PDAC and CP, untargeted LC-MS was applied to plasma samples. Contrary to the present study, clinical data on e.g. disease stage and smoking status was available, which is important to avoid known confounding factors; however, the sample size (n = 10) was very limited which makes the results less generalizable. Other studies have applied different analytical metabolomics platforms to discriminate PDAC and CP in bodily fluids. In a study using gas chromatography-mass spectrometry, a majority of the serum discriminative metabolites were amino acids (Kobayashi et al. 2013), but included also some metabolites that would not have been particularly well captured in our LC-MS set-up such as some sugar species including, arabinose, ribulose and 1,5-Anhydro-D-glucitol and some organic acids such as uric acid, nonanoic acid and caprylic acid. In the mentioned study, CP samples were however included in the validation cohort only. Amino acids were also connected to future risk of pancreatic cancer in a metabolomics study applying LC-MS to prediagnostic plasma (Mayers et al. 2014). In the present study a number of amino acids were found dysregulated in PDAC compared to CP in the validation cohort only (data not shown), but lacking confirmation in a second cohort these results were excluded. Further, two studies have used nuclear magnetic resonance to study plasma and urine samples, respectively, but have not validated their results in a second cohort (Zhang et al. 2012) or had only three CP samples out of a total of 25 samples included in the benign control group (Davis et al. 2013).

In the present study, the levels of a number of phospholipids were altered in PDAC compared to CP in both cohorts. Phospholipids were also found dysregulated in previous studies (Urayama et al. 2010; Ritchie et al. 2013; Sakai et al. 2016). However, we excluded all phospholipids as potential markers since they were down-regulated in PDAC in the discovery cohort as opposed to up-regulated in the validation cohort (Figs. 2, 4c, d). A possible explanation for these results is that different blood sample matrices, i.e. serum and plasma, were used in the two cohorts. Serum and plasma samples from the same individual are known to display different concentration levels for some metabolites, including phospholipids, due to the differences in sample handling (Yu et al. 2011). Noteworthy, as systemic phospholipid levels are altered by inflammatory responses in general, they are unlikely candidates for disease specific markers (Lindahl et al. 2016).

As mentioned above, the two bile acids glycocholic acid and chenodeoxyglycocholate were significantly increased in PDAC compared to CP in the present study (Figs. 3, 5b). A possible explanation for increased levels of circulating bile acids is tumor growth into the bile duct. Another theory includes bile acid reflux into the pancreas, leading to pancreatitis and eventually malignant cell transformation; a direct carcinogenic effect of bile acids is also possible (Feng and Chen 2016). Bile acid levels in prediagnostic plasma have also been connected to future risk of pancreatic cancer (Mayers et al. 2014), supporting our results and reasoning.

Two of the discriminative metabolite markers identified here, N-palmitoyl glutamic acid and hexanoylcarnitine (Figs. 3, 5b), are fatty acid conjugates with an amino acid and carnitine, respectively. N-acyl amino acids were only recently discovered as a novel group of compounds and little is known about their function (Hanus et al. 2014). However, a recent study in mice showed that certain N-acyl amino acids regulate cell metabolism through mitochondrial uncoupling (Long et al. 2016). Acylcarnitines in turn are vital for the transport of fatty acids into the mitochondrial matrix (Indiveri et al. 2011). Short-chain acylcarnitines including hexanoylcarnitine were previously found increased in serum in type 2 diabetes compared to normal glucose tolerance (Mai et al. 2013); both CP and PDAC may cause diabetes (Pinho et al. 2014).

The levels of the nitrogenous metabolite PAGN were also increased in PDAC compared to CP in the present study (Fig. 5b). PAGN is a product of gut microbiota-host co-metabolism found in urine and blood (Barrios et al. 2015), and there are indications that the gut microbiota has an important role in carcinogenesis (Schwabe and Jobin 2013).

In conclusion, this work has identified five metabolites capable of discriminating PDAC and CP in blood either as single or as a panel of markers. Findings were validated in a separate patient cohort. To determine the potential clinical benefit of these markers, further evaluation in larger clinical studies is needed. Nevertheless, we believe that these metabolites are potentially useful for early diagnosis of PDAC in CP patients.

## Electronic supplementary material

Below is the link to the electronic supplementary material.

Supplementary material 1 (DOCX 302 KB)

Supplementary material 2 (DOCX 295 KB)

Supplementary material 3 (DOCX 41 KB)

Supplementary material 4 (DOCX 11 KB)

## Abbreviations

RPLC-MS: Reversed phase liquid chromatography coupled to mass spectrometry
PDAC: Pancreatic ductal adenocarcinoma
CP: Chronic pancreatitis
OPLS-DA: Orthogonal partial least squares discriminant analysis
