# Supplementary material for: Discrimination of pancreatic cancer and pancreatitis by LC-MS metabolomics
Source: Metabolomics. 2017 Apr 1;13(5):61. doi: 10.1007/s11306-017-1199-6 (PMC5376388; doi:10.1007/s11306-017-1199-6)
Supplement: Supplementary file 1 — Supplementary material 1 (DOCX 302 KB) [file 11306_2017_1199_MOESM1_ESM.docx]

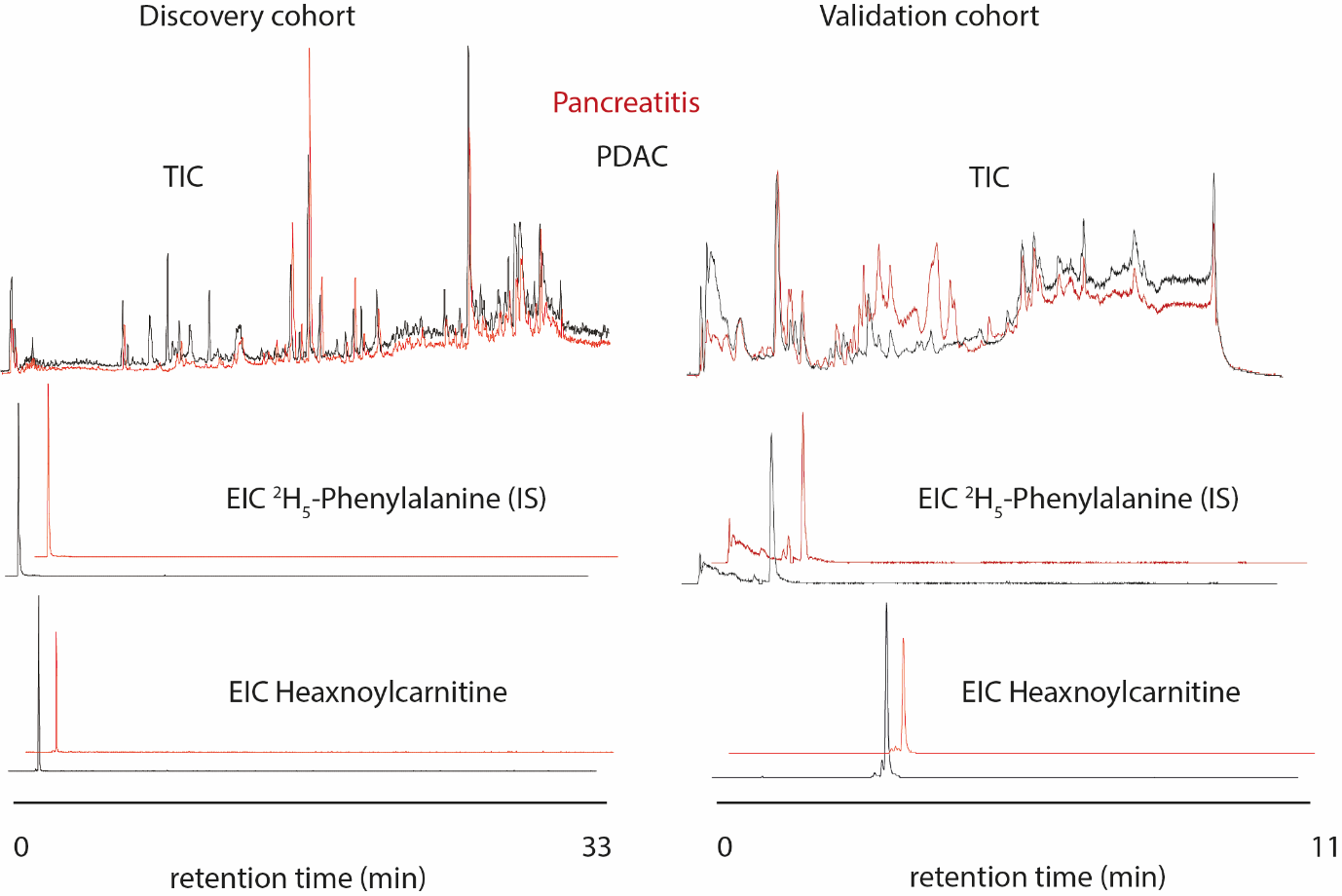


**Supplemental figure 1.** Example of total ion chromatograms (TIC) and extracted ion chromatograms (EIC) for PDAC (black) and pancreatitis (red) from the discovery and validation cohorts. The EIC of phenylalanine-IS and hexanoyl-carnitine are slightly off axis for increased visability of feature intensity.
