# Supplementary material for: Discrimination of pancreatic cancer and pancreatitis by LC-MS metabolomics
Source: Metabolomics. 2017 Apr 1;13(5):61. doi: 10.1007/s11306-017-1199-6 (PMC5376388; doi:10.1007/s11306-017-1199-6)
Supplement: Supplementary file 2 — Supplementary material 2 (DOCX 295 KB) [file 11306_2017_1199_MOESM2_ESM.docx]

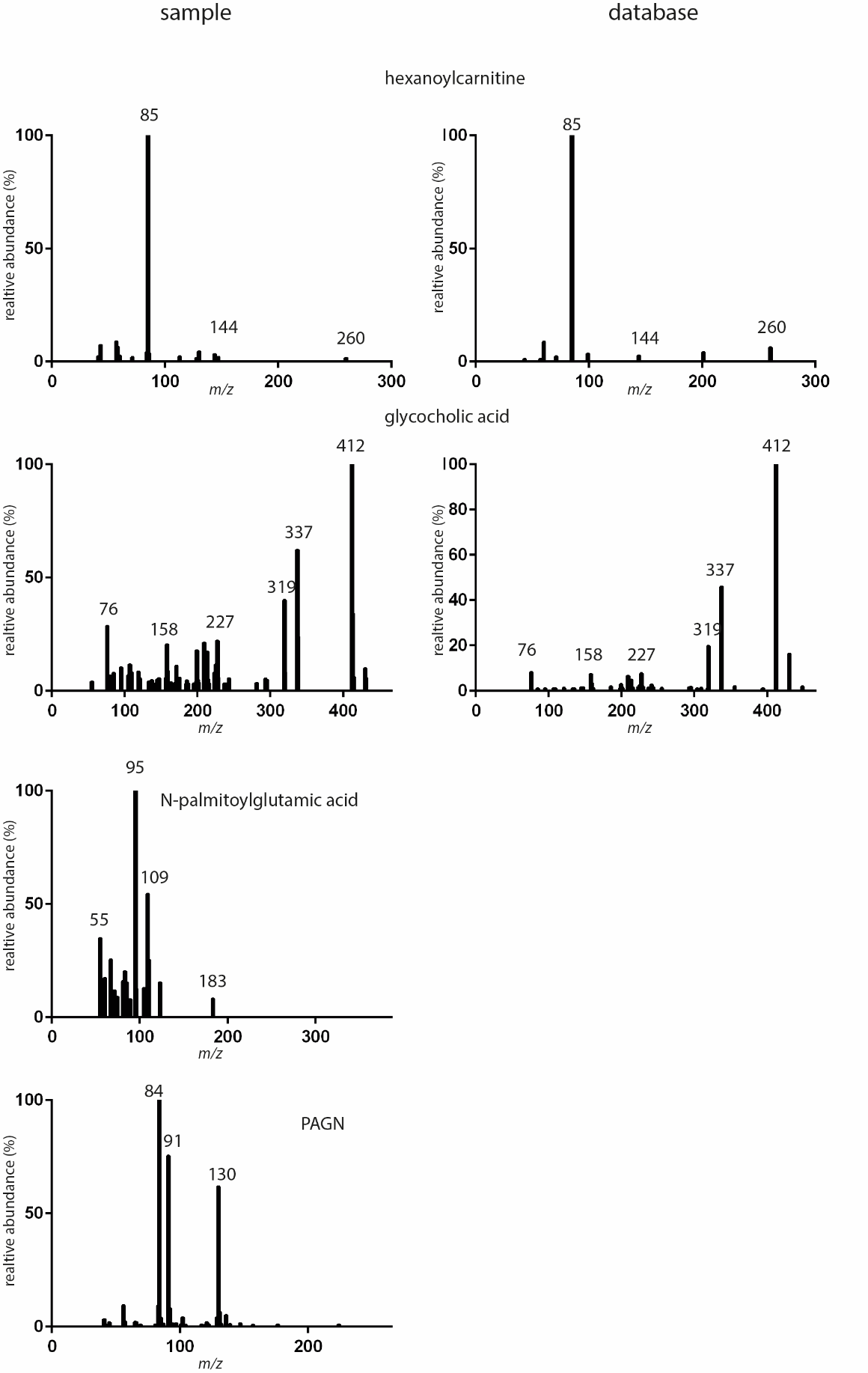


**Supplemental figure 2.** MS/MS spectra for identified compounds. For two of the compounds no database spectra was available.
