# Supplementary material for: Discrimination of pancreatic cancer and pancreatitis by LC-MS metabolomics
Source: Metabolomics. 2017 Apr 1;13(5):61. doi: 10.1007/s11306-017-1199-6 (PMC5376388; doi:10.1007/s11306-017-1199-6)
Supplement: Supplementary file 3 — Supplementary material 3 (DOCX 41 KB) [file 11306_2017_1199_MOESM3_ESM.docx]

a)

b)

Supplementary figure 3. (a) PCA score (PC1 vs PC2) plot based on the 4578 variables identified using XCMS processing. A batch effect is observable indicating that there is noise in the data and that supervised classification (OPLS-DA) should be used for further processing. However, samples from both groups are being present in all clusters suggesting that all variance not originate in noise. (b) Score plot of PC2 vs PC3, noise effect not visible. Two potential outliers, further investigation as of why these would be outliers resulted in that these samples were kept in the analysis.
