# Supplementary material for: Discrimination of pancreatic cancer and pancreatitis by LC-MS metabolomics
Source: Metabolomics. 2017 Apr 1;13(5):61. doi: 10.1007/s11306-017-1199-6 (PMC5376388; doi:10.1007/s11306-017-1199-6)
Supplement: Supplementary file 4 — Supplementary material 4 (DOCX 11 KB) [file 11306_2017_1199_MOESM4_ESM.docx]

| **Stable isotope labelled Phenylalanine (IS)** | **Discovery cohort** | **Validation cohort** |
| --- | --- | --- |
| Average integrated area | 22089856 | 1929175 |
| Standard deviation of integrated area | 2574429 | 359622 |
| CV (%) | 11.6 | 18.6 |

**Supplementary table 1.**

Coefficient of variation for internal standard in discovery and validation cohorts
